# Missed presentation of Crohn's disease in a patient with a fistulating thigh abscess: a case report

**DOI:** 10.1186/1757-1626-3-26

**Published:** 2010-01-14

**Authors:** Deborah Ferguson, Aravind Suppiah, Eleanor Richards, Jeremy Wilson, Deepak Pai, Asit Kar

**Affiliations:** 1 Scunthorpe General Hospital, Cliff Gardens Cliff Gardens, Scunthorpe, South Humberside DN15 7BH, UK

## Abstract

**Background:**

Musculoskeletal presentations of Crohn's disease are rare and they include psoas abscess, thigh abscesses and in extreme cases septic arthropathy.

**Case presentation:**

Herein, we present a 53 year old gentleman with bilateral thigh fistulae discovered to be a new diagnoses of extra-intestinal Crohn's disease

**Conclusion:**

It is important to consider Crohn's disease in patients that present with unusual or persistent fistulae and to consider this essential when there are atypical organisms present.

## Background

Crohn's disease (CD) is a granulomatous inflammatory condition affecting the gastrointestinal tract with an incidence of 1-3 per 100,000 [1]. It usually presents with gastrointestinal or non-specific symptoms such as weight loss. CD is associated with an array of extra-intestinal manifestations include eyes (uveitis, iritis), skin (erythema nodusum, pyoderma ganrenosum), cardiorespiratory (interstitial lung disease, pericarditis) and prolithogenic conditions (chole- or nephrolithiasis). Musculoskeletal involvement is less common and typically involves sero-negative non-deforming polyarthropathy or ankylosing spondylitis.

A hallmark of CD is fistula formation which occurs in 17-50% of patients, usually between bowel, abdominal viscera or to abdominal wall or perineum [2]. Fistulation into soft tissue or peri-articular regions are unusual. Psoas abscesses are rare but a documented presentation of CD [3]. Musculoskeletal fistulas are extremely rare with only 3 reported cases to our knowledge of hamstring [4] and gluteal abscesses [5]. Additionally, none of these cases report these as first presentations of CD, but rather complications in patients with already known disease.

We present a unique case of patient with previously unknown CD, presenting for the first time with a discharging right hip abscess in the absence of gastrointestinal symptoms. We discuss the subsequent diagnostic pitfalls leading to delayed diagnosis, and put identify warning features that should alert any surgeon as to this unique presentation of CD, and also CD as possible aetiology in musculo-skeletal pathology [6].

## Case presentation

**Figure 1:**
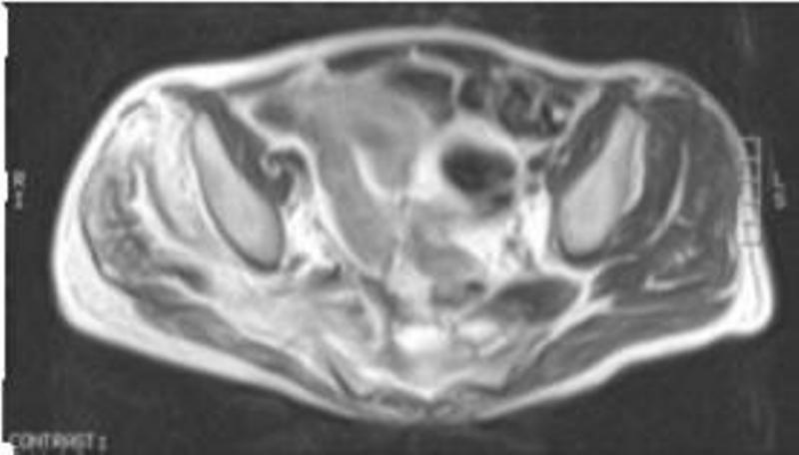
**CT showing gas in the bladder and two locules of gas in the soft tissue just outside piriformis muscle and adjacent to the right femur**.

**Figure 2:**
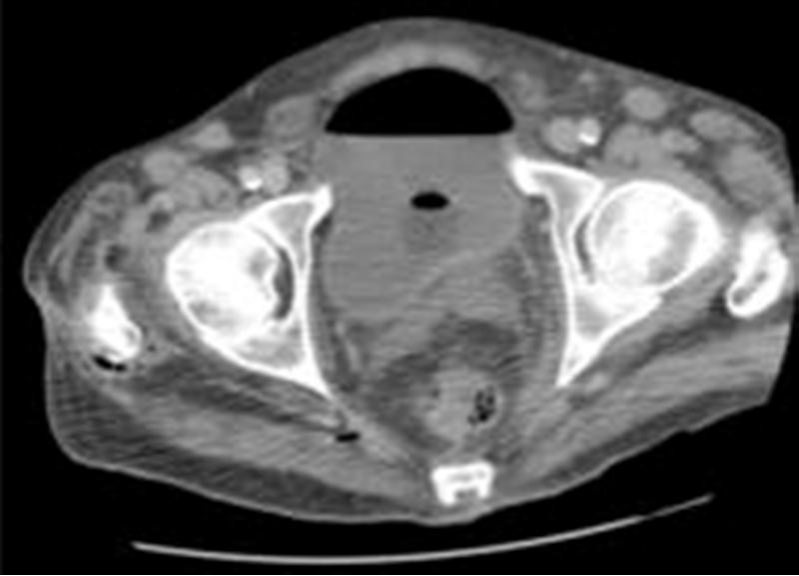
**STIR axial of the pelvis showing oedema with in the soft tissue involving the right piriformis muscle extending into the gluteal muscles on right side**.

A 53-year-old white British gentleman presented with a 2 month history of purulent discharge from a sinus on the right lateral thigh. He was otherwise systemically well and has no significant past medical history. On admission he was apyrexial and haemodynamically stable. Examination revealed normal right hip movement. X-ray showed soft tissue radiolucent abnormality just distal to the lesser trochanter. Inflammatory markers were raised (CRP 165, WCC 13.1 × 10^9^/L and neutrophils10.5 × 10^9^/L) and wound cultures grew group B Streptococci and he was commenced on flucloxacillin and benzyl penicillin. Limited-view right hip MRI showed two locules of gas in the soft tissue just outside piriformis muscle and adjacent to the right femur (Figure 1) and signal changes in the right gluteal muscles (Figure 2) and a small collection within the muscles lateral to the right hip joint with no evidence of osteomyelitis.

He was referred to orthopaedics where right hip abscess exploration with washout and primary closure was performed and the patient discharged the following day. He re-presented a week later with recurrent discharge from the same site, which was re-explored but on this occasion left open. He underwent further washout with closure 3 days later. The discharging "sinus" recurred again within 4 days. On this occasion, the wound was left open and left to heal by secondary intention. Plastics opinion was sought due to complex nature of the lesion and already extensive management. This complicated thigh wound was managed with interval washouts and long-term antibiotics.

Five years later, the patient was referred to general surgical clinic with presumed left leg cellulitis. He still had a chronic right-sided discharging sinus. Abdominal CT revealed a vesico-ileal fistula with thickened bowel wall and gas pockets in the left poster-medial thigh and the right proximal femoral and para-sacral spaces, suggestive of fistulating CD. The following day, right hemicolectomy, excision of ileo-vesical fistula and drainage of left thigh abscess was performed. Post-operative histology confirmed CD. The patient was discharged after 24 days requiring no treatment for CD on discharge and was follow-up by gastroenterologist. To date the patient remains well with no evidence of further soft tissue injury.

## Discussion

Psoas abscess is a rare but recognised complication of CD. Thigh sinuses and periarticular collections are extremely rare and to our knowledge there are only 3 reported cases of thigh disease and 2 reports of CD leading to septic arthritis, one of whom died. In both cases, the patient was already known to have CD [6]. This is the first case of undiagnosed CD presenting as initially bilateral thigh abscesses which caused diagnostic and hence, subsequent therapeutic dilemma lasting several years.

Diagnosis was delayed due to the absence of colorectal symptoms and the inability to visualise a communicating entero-cutaneous fistula on limited view right hip MRI. The patient then presented with a further contra lateral cellulitis, suggesting underlying infection from which enterococcus species was grown. Urgent surgical referral was sought and the patients went on to have curative surgery and remains symptom free at 1 year.

## Conclusion

In the absence of a clear source or septic focus for hip abscess or septic arthritis of the hip, early gastroenterology opinion must be sought, even in the absence of colorectal symptoms, to avoid delays in diagnosis and treatment. This becomes more urgent in the presence of later warning signs which are; recurrence of peri-articular collections despite washouts, the development of a contra-lateral collection or presence of atypical organisms.

## Consent

Written informed consent was obtained from the patient for publication of this case report and accompanying images. A copy of the written consent is available for review by the Editor-in-Chief of this journal.

## Competing interests

The authors declare that they have no competing interests.

## Authors' contributions

DF reviewed the patients case notes, drafted the initial and final copy of the case report and completed submission after reading and approving final manuscript. AS reviewed the patients case notes and was a major contributor in writing and then reading and approving final manuscript. He also gained patient consent. ER reviewed the manuscript, reviewed, submitted the radiological imaging and read and approved the final manuscript. JW read and approved the final manuscript. DP reviewed and assisted with submission of the radiological imaging alongside reading and approving final manuscript. AK reviewed the patients case notes and reviewed and read and approved the final manuscript.
